# Leveraging genomic sequencing data to evaluate disease surveillance strategies

**DOI:** 10.1016/j.isci.2023.108488

**Published:** 2023-11-19

**Authors:** Benjamin Anderson, Derek Ouyang, Alexis D’Agostino, Brandon Bonin, Emily Smith, Vit Kraushaar, Sarah L. Rudman, Daniel E. Ho

**Affiliations:** 1 Stanford University, Stanford, CA 94305, USA; 2 County of Santa Clara Public Health Department, San Jose, CA 95126, USA; 3 Theiagen Genomics, Highlands Ranch, Colorado 80129, USA; 4 California Department of Public Health, Sacramento, CA 95814, USA

**Keywords:** Public health, Virology

## Abstract

In the face of scarce public health resources, it is critical to understand which disease surveillance strategies are effective, yet such validation has historically been difficult. From May 1 to December 31, 2021, a cohort study was carried out in Santa Clara County, California, in which 10,131 high-quality genomic sequences from COVID-19 polymerase chain reaction tests were merged with disease surveillance data. We measured the informational value, the fraction of sequenced links surfaced that are biologically plausible according to genomic sequence data, of different disease surveillance strategies. Contact tracing appeared more effective than spatiotemporal methods at uncovering nonresidential spread settings, school reporting appeared more fruitful than workplace reporting, and passively retrieved links through survey information presented some promise. Given the rapidly dwindling cost of sequencing, the informational value metric may enable near real-time, readily available evaluation of strategies by public health authorities to fight viral diseases beyond COVID-19.

## Highlights

• We analyzed 10,131 high-quality SARS-CoV-2 viral sequences from Santa Clara County
• Informational value is the fraction of sequenced links with plausible transmission
• Contact tracing and spatiotemporal clustering both yielded high informational value
• School, long-term care facility, and jail reporting yielded more than at workplaces

## Graphical abstract

**Figure:**
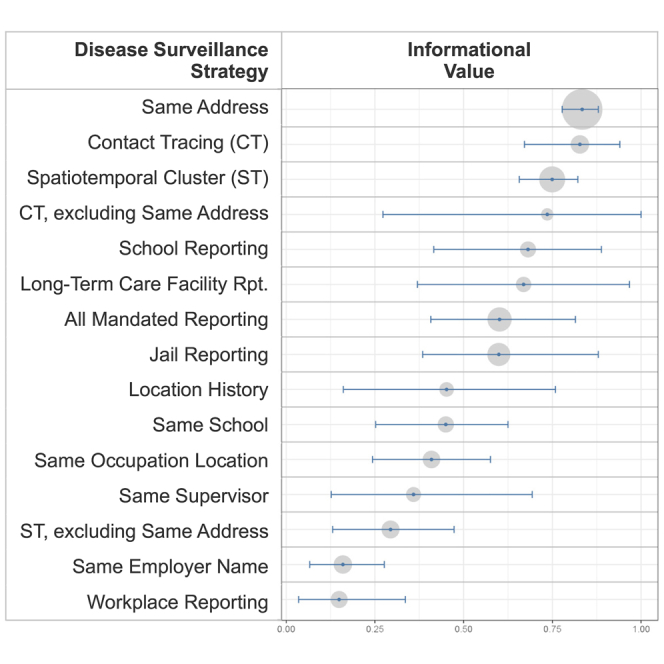

## Introduction

One of the central questions in public health is the effectiveness of distinct disease surveillance strategies.^1^ In the severe acute respiratory syndrome coronavirus 2 (SARS-CoV-2) pandemic, officials have proposed, deployed, and terminated a wide range of techniques. State and local public health authorities, for instance, have prioritized testing in congregate settings (e.g., long-term care facilities [LTCFs], shelters, and jails), employed contact tracing in conjunction with positive test notification, and directed mandated reporting and outbreak investigation in schools, workplaces, and congregate settings. Emerging tools, such as spatiotemporal scan statistics for identifying clusters of interest, have also attracted interest.^2^ These approaches have been used for many communicable diseases, from foodborne illness to sexually transmitted infections,^3^^,^^4^ but there is limited evidence as to the relative effectiveness of distinct disease surveillance strategies, particularly as a disease spreads.

In Santa Clara County (SCC), California, home to over 1.8 million residents, the Public Health Department developed one of the largest COVID-19 case investigation and contact tracing initiatives in the country, along with myriad approaches to monitor and address potential disease spread. We briefly review principal strategies as deployed or considered in SCC during the study period from May 1 to December 31, 2021. First, individualized *contact tracing* has been widely adopted to map and monitor community transmission of SARS-CoV-2.^5^ Contact tracing has been used in the past for surveillance of a wide variety of diseases, including smallpox and SARS, and aims to stem COVID-19 outbreaks by identifying the close contacts of diagnosed cases and informing them of their potential exposure to the virus.^6^ Studies of the efficacy of COVID-19 contact tracing have variously found associations or insignificant or inconclusive effects on infections, secondary attack rate, hospitalizations, deaths, and effective reproduction number.^7^^,^^8^^,^^9^^,^^10^ A systematic review of the empirical evidence for contact tracing for several diseases found that it can be an effective public health tool but noted a “scarcity of quantitative evidence on its effectiveness” with wide-ranging practices.^11^

Second, *mandated reporting* has been employed by public health authorities where extensive identification, testing, querying, and monitoring of individuals, who were exposed to a first positive case and might be part of a larger outbreak, can be targeted at the facility level with the cooperation of designated third parties. SCC and other California counties mandated school and workplace outbreak reporting through a state portal to facilitate local outbreak investigations to varying degrees throughout the pandemic.^12^^,^^13^^,^^14^ Standardized and comprehensive reporting by employers may expose workplace-specific risks that individualized contact tracing efforts miss and may inform workplace- or industry-specific nonpharmaceutical interventions.^15^ SCC delegated one distinct team to perform contact tracing through interaction with a case or their family, and another team to perform in-depth outbreak investigations, working with COVID designees from an involved institution (e.g., school, employer, and congregate facility) to generate a curated cohort of potentially exposed individuals and to actively increase their testing rate. While various forms of mandated reporting and associated outbreak investigation have been widely deployed for COVID-19 control, they are time-consuming and pose a variety of challenges related to responsiveness, confidentiality, and liability.^16^^,^^17^ Much of the academic literature consists of case studies of individual investigations rather than evidence for the utility of reporting or investigation as a public health tool.^18^^,^^19^

Third, *spatiotemporal clustering*, which identifies case clusters based on proximity in space and time, has also been proposed as a COVID-19 surveillance strategy. Spatiotemporal clustering has long been a component of syndromic surveillance tools and platforms, such as the Centers for Disease Control and Prevention (CDC) Electronic Surveillance System for the Early Notification of Community-Based Epidemics (ESSENCE) and Early Aberration Reporting System (EARS), which ingest syndromic surveillance data and provide alerts and visualization for spatiotemporal clusters of interest.^20^ In the context of COVID-19 surveillance, spatiotemporal methods may facilitate the detection of clusters beyond a single household, and can further refine cluster detection by measuring the statistical significance of a cluster relative to the background case rate. COVID-19 spatiotemporal clustering analyses have most commonly utilized SaTScan; for instance, Greene et al. (2021) use the SaTScan software package to identify local hotspots of COVID-19 cases in New York at the census tract level.^2^ Similar work analyzing COVID-19 case data with space-time scan statistics has been conducted across the US and in other countries, including Spain and Brazil.^21^^,^^22^^,^^23^

One common challenge around assessing these disease surveillance strategies is validation. Confirming a transmission link is resource-intensive, and, even with such information, it is hard to assess how counterfactual strategies might have fared. In this paper, we show that sequenced viral RNA, collected at an unprecedented scale during the COVID-19 pandemic, provides opportunities to assess the relative ability of disease surveillance strategies to recover related cases in near real time as the disease spreads. While their primary use has been to track and detect attributes of emerging variants,^24^^,^^25^ genomic data can aid to more systematically investigate how reliably different disease surveillance strategies uncover chains of disease transmission.^16^^,^^26^^,^^27^^,^^28^ In addition, such data may help assess strategies that have not yet been widely implemented, such as spatiotemporal clustering or automatic case linkage using information like workplace location.

In the face of scarce public health resources, it is critical to understand which disease surveillance strategies provide reliable transmission information, what underutilized strategies may exist, and how strategies may serve as complements or substitutes. We hence leverage rich linked datasets from SCC and illustrate one approach for comparing the relative ability of disease surveillance strategies to identify related cases by utilizing increasingly available data from whole-genome sequences. While our illustration is with SARS-CoV-2 data, the approach can be used with any communicable disease that yields genomic data.

## Results

**Figure 1:**
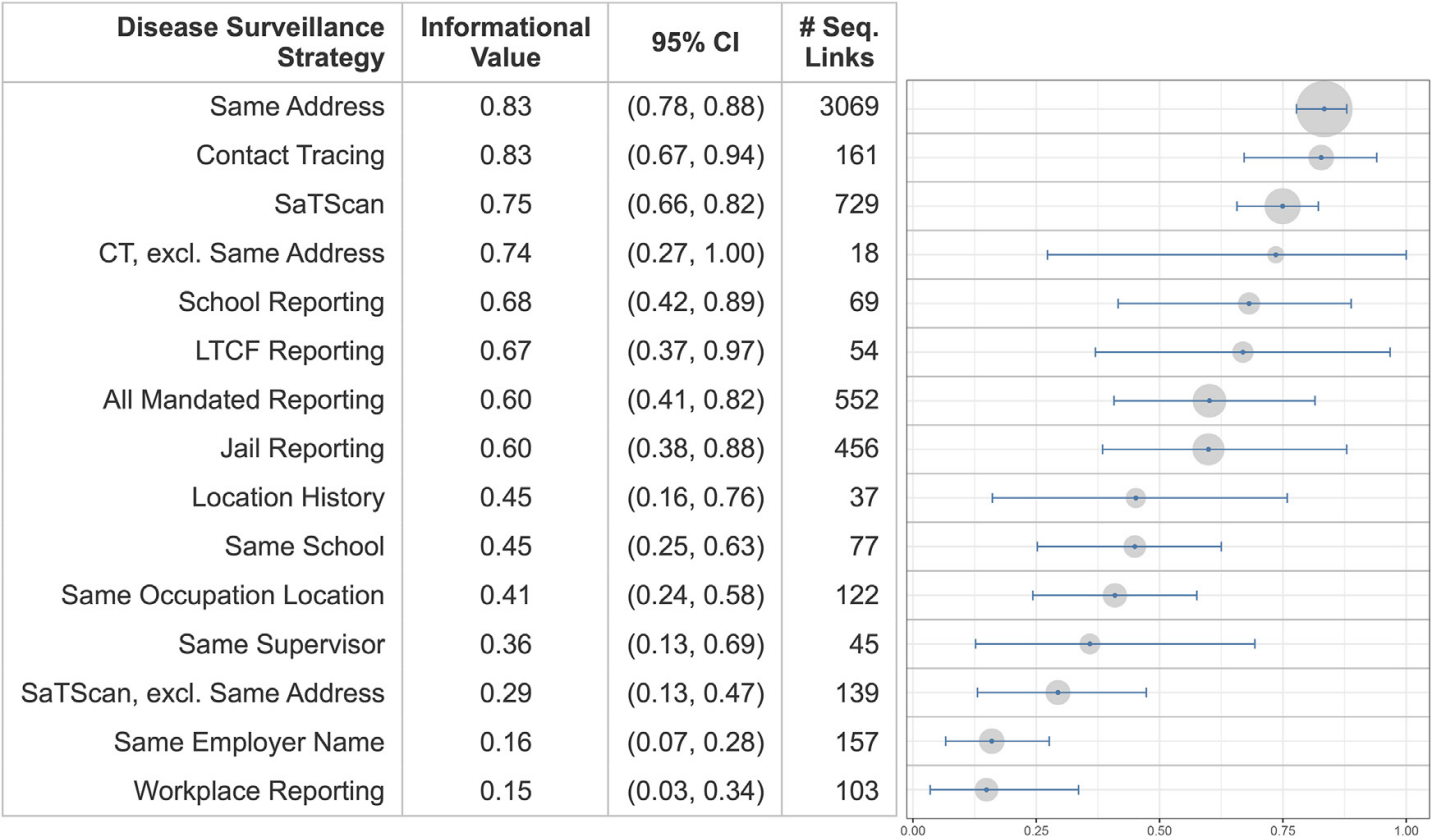
Informational value of disease surveillance strategies Author’s analysis of Santa Clara County CalCONNECT and genomic sequencing data from May 1 to December 31, 2021. For each disease surveillance strategy, we show: the informational value (proportion of sequenced case pairs which are plausible transmission links according to whole-genome sequence data); the 95% confidence interval on informational value; and the number of proposed links which are sequenced. *Contact Tracing*: identified as close contacts by contact tracing. *Same Address*: shared home address. *SaTScan*: assigned to the same cluster by spatiotemporal analysis. *All Mandated Reporting*: linked through mandated reporting in a high-risk setting; *School*, *Long-Term Care Facility*, *Jail*, and *Workplace Reporting* are sufficiently numerous and sequenced to also be presented on their own. The following five strategies are based on matching results from the case interview or survey. *Location History*: matching location visited within the same time period. *School*: same school name, school type, and school location. *Occupation Location*: same workplace location, usually an address. *Supervisor*: same supervisor name, phone number, or e-mail. *Employer Name*: same employer name. CI, confidence interval; CT, contact tracing; LTCF, long-term care facility. See also Figures S1–S4.

We examine the effectiveness of existing and potential strategies for identifying linked cases, summarized in Figure 1. Overlap counts and p values between all pairs of strategies are shown in Figures S1 and S2 in the supplemental information (SI), respectively.

### Contact tracing and spatiotemporal clustering

We find that a high proportion of close contacts (83% [95% confidence interval, 67–94%]) identified by contact tracing are plausible transmission links according to whole-genome sequence data. However, a key question is whether contact tracing provides any marginal informational value compared to same address links (83% [78–88%]) which can be passively determined from the original laboratory results, thereby not requiring contact tracing to uncover. Notably, the 17% of close contacts links that do not share an address (i.e., the non-obvious relationships we might depend on contact tracers to find) are also highly plausible (74% [27–100%]), but infrequent. We also find that spatiotemporal links via SaTScan yield high informational value (75% [66–82%]), but when same address links are excluded, leaving only 45% remaining proximate connections, informational value drops (29% [13–47%]), suggesting a lower likelihood of direct transmission.

### Mandated reporting and passive information

Same address linkage provides higher informational value (p < 0.01) than mandated reporting (60% [41–82%]) as a whole, but when disaggregated into separate categories, school (68% [42–89%]), jail (60% [38–88%]), and LTCF reporting (67% [37–97%]) yield relatively plausible transmission links in contrast (p < 0.01) to workplace reporting (15% [3–34%]). Passively retrieved links of information, as volunteered by individuals during the contact tracing interview or survey instead of as submitted by mandated reporters, generally have lower informational value than active strategies, but present some promise. Matching location history (45% [16–76%]) and school (45% [25–63%]) perform moderately well. Passively collected occupation location (41% [24–58%]) outperforms (p < 0.01) the active efforts of workplace reporting in finding plausible transmission. We find that the granularity of information collected makes a difference, as grouping cases by more specific employment details like occupation location (p < 0.01) or shared supervisor name, phone number, or e-mail (36% [13–69%], p = 0.08) yields a higher proportion of plausible links than simply grouping by employer name (16% [7–28%]).

## Discussion

Our initial assessment has yielded valuable insights about SCC’s range of disease surveillance strategies. School reporting appears more fruitful than workplace reporting, perhaps in part due to the greater and more relevant training of school COVID designees, often professional school nurses, compared to that of workplace designees, often offsite human resources staff. Our results may point to important differences between not only disease surveillance strategies, but also the underlying spread settings in which surveillance is conducted (i.e., classroom and office layouts). Contact tracing yields many potential links through close contacts, but 83% of the recovered links were from the same home address. The relatively high informational value of close contacts outside the home, compared to more data-driven spatiotemporal attempts at the same, suggests the promise of contact tracing protocols that focus more on uncovering nonresidential spread settings, where circumstantial evidence is otherwise low. Moreover, our analysis suggests there are lighter-weight passive alternatives, such as automated linkage of shared location histories, schools, and workplaces via surveys, which may potentially serve as time-saving substitutes or precursors to active outbreak investigation. We note that all of these examined fields were designed as open text fields, and that the true potential of passive surveillance may hinge on improved user interface design (e.g., autocomplete, drop-down lists, and entity resolution).

### Limitations of the study

There are several limitations to our approach. First, while sequencing is broadly representative of the county population, it was not designed as a random sample. The sequencing rate varies across disease surveillance strategies, in part because availability of specimens for genomic sequencing was often tied to the County hospital system or testing sites, which overrepresent portions of the county with higher social vulnerability, and in part because the County explicitly targeted testing in high-risk congregate settings like jails to varying degrees throughout the pandemic. Our comparisons of informational value could be confounded if sequencing practices bias toward transmissibility within specific spread settings. However, in practice, SCC could not operationally bias sequencing within disease surveillance strategies; rather, the County could only vary the overall availability of sequencing across strategies, which we expect to only affect the precision of our results.

Another potential source of selection bias is differences between the two sources of genomic data we rely on in our study, which flow through very different chains of custody. COVID-19 tests that are analyzed by the SCC Public Health Laboratory are sequenced in-house, and then submitted to the County’s PCR test-based repository of SARS-CoV-2 genomes, Terra,^29^ for analysis. Tests that are performed by Fulgent are sequenced at their own laboratories, submitted to the CDC, and published in the Global Initiative on Sharing Avian Influenza Data (GISAID), a public database of SARS-CoV-2 and influenza sequences,^30^^,^^31^^,^^32^ with separate metadata including personal identifiers shared privately with the County. Both sequencing pipelines were crucial to the County’s COVID-19 response, but since they are independent, unobserved practices of the third-party laboratory could introduce bias. As a robustness check, in Figures S3 and S4 in the SI, we present informational value broken out by the source of genomic data. For instance, for Terra, we subset to the links for which both cases are sequenced by Terra. We exclude congregate setting investigations (including jails and LTCFs) because tests from these disease surveillance strategies were nearly entirely sequenced by the County. While some variation exists at the strategy level between the two sources of sequencing, on the whole they present a similar rank order of informational value. The Pearson correlation of informational value between Terra and GISAID is 0.84, and the Spearman correlation is 0.83. To the extent that our findings could still be driven in other ways by selection bias, public health authorities considering the use of similar sequence-based assessment methods should assess the representativeness of their sequencing and explore techniques like uncertainty sampling to improve sequencing balance across strategies.^33^^,^^34^

Second, disease surveillance strategies are not monolithic. SCC designed its contact tracing to prioritize ZIP codes with a higher social vulnerability index and disproportionately invested resources toward school reporting and investigations to help ensure schools stayed open, so SCC’s results for these strategies may not generalize to contact tracing and mandated reporting in other jurisdictions. Disease surveillance strategies were not even consistent within SCC; the definition of which students should be included in school outbreak reporting varied by school and over time, from “only those not wearing masks in the classroom” to “the whole classroom” to “all classmates, even those who were not at school.” While we are able to generally compare informational yield across disease surveillance strategies, our sample sizes limit us from understanding the specific, potentially manageable conditions (e.g., reporting protocol, room size, speed of testing, and improved form collection) under which low-yield strategies may turn out to excel. Future work can readily assess the informational value of disease surveillance strategy variations beyond SCC’s, and with greater granularity, using increasingly available viral sequencing data.

Third, our results inform, but should not be interpreted as conclusive of, the optimal strategy. The resources required to implement different surveillance strategies—ranging from monetary to technological to political—may vary more widely than their ability to identify plausible transmissions. The same is true of the varied cost of *not* implementing different strategies. Informational value may in fact directly tradeoff with resource investment; for instance, congregate setting investigations may have been more extensive in testing of full populations, hence yielding lower genomic similarity, because of the higher risks of, and political impetus to avoid, transmission in those vulnerable populations. Any policy choice should incorporate the relative benefits and costs. At its core, higher informational value means a lower likelihood of “wrong links,” which are undesirable because they incur significant costs in both public health resources and impacts on livelihoods, i.e., someone’s ability to earn income, provide childcare, etc.

Last, the efficacy of disease surveillance strategies would be expected to be disease-specific (i.e., contact tracing may be unrealistic for diseases with significantly higher airborne transmissibility). In the case of SARS-CoV-2, genomic similarity is itself limited due to the relatively slow mutation rate and the potential for parallel evolution events^24^^,^^35^; nonetheless, our approach yielded substantive insights.

### Conclusion

COVID-19 led to a flurry of distinct strategies to investigate and identify cases, far outpacing the bandwidth to assess their relative usefulness. While pandemic response to COVID-19 has receded, our results illustrate an important lesson that carries relevance for public health prospectively. Our approach illustrates how public health authorities might begin to validate distinct disease surveillance strategies using genomic sequences. Given the rapidly dwindling cost of genomic sequencing, the informational value metric may open the door to not just variant tracking, but also near real-time, readily available evaluation of strategies to fight many viral diseases beyond COVID-19 (e.g., Mpox). Our investigation adds important evidence on which disease surveillance strategies appear to be effective in the face of significant public health investments.

## STAR★Methods

### Key resources table

**Table:** 

REAGENT or RESOURCE	SOURCE	IDENTIFIER
**Deposited data**		

152,337 sequences available up to February 24, 2022, subsequently filtered and matched to individual COVID-19 patients from Santa Clara County, totaling 10,131 sequences.	Global Initiative on Sharing Avian Influenza Data (GISAID) COVID-19 database^30^^,^^31^^,^^32^	https://doi.org/10.55876/gis8.221223tc
Locations in the SARS-CoV-2 genome that have been identified as problematic for analysis for various reasons.	Turakhia et al.^36^	https://github.com/W-L/ProblematicSites_SARS-CoV2/blob/master/problematic_sites_sarsCov2.vcf

**Software and algorithms**		

SaTScan, v.10.0.1	Kulldorf^37^	https://www.satscan.org
Terra, IQ-TREE	Nguyen et al.^38^	https://app.terra.bio
Phylogenetic bootstrap	Felsenstein^39^	https://doi.org/10.2307/2408678

**Other**		

Aggregate data, the study protocol, and code	This paper	https://github.com/reglab/covid-19-disease-surveillance

### Resource availability

#### Lead contact

Requests for further information should be directed to and will be fulfilled by the Lead Contact, Daniel E. Ho (deho@stanford.edu).

#### Materials availability

This study did not generate new materials.

#### Data and code availability

This paper analyzes existing, publicly available sequences, but due to privacy concerns, linkages to patient-level data (obtained through SCC’s contact tracing database, CalCONNECT) cannot be shared. A DOI containing accession numbers from an initial superset of sequences is available in the key resources table.

Other aggregate data, the study protocol, and code have been deposited at GitHub and are publicly available as of the date of publication. The repository link is available in the key resources table.

Any additional information required to reanalyze the data reported in this paper is available from the lead contact upon request.

### Experimental model and study participant details

This study follows Strengthening the Reporting of Observational Studies in Epidemiology (STROBE) reporting guidelines for cohort studies and examines COVID-19 case investigation and contact tracing and genomic sequencing data from existing databases used by public health jurisdictions. The SCC Public Health Department and Stanford University, Stanford, California, deemed the work public health surveillance; the Revised Common Rule deems “public health surveillance activities,” including testing necessary to monitor disease outbreaks by a public health authority, not subject to Institutional Review Board (IRB) oversight under 45 CFR § 46. Thus, it was not submitted for IRB approval, but was subjected to privacy and compliance review by SCC. Participation in this study was voluntary: COVID-19 testing was undertaken only when permission was granted. Our study period ranges from May 1 to December 31, 2021, a period which encompasses the COVID-19 surge from the Delta variant during the summer and the beginning of the surge from the Omicron variant in the winter. We obtain 67,364 COVID-19 patient case records from SCC’s contact tracing database (CalCONNECT), which comprises deduplicated person records and incidents for each episode of COVID-19 disease and/or identification as a contact to COVID-19. The case records are pre-populated with patient name, date of birth, and home address from the laboratory which conducted the polymerase chain reaction (PCR) test, and subsequently augmented with other patient-level attributes through an automated survey and/or phone interview with a contact tracer. Table S1 in the SI contains gender, race, and ethnicity information for study participants.

### Method details

#### Disease surveillance strategies

CalCONNECT provides information about two primary existing disease surveillance strategies of interest. First, in *contact tracing*, close contacts reported by a positive case may be subsequently contacted by the contact tracer and encouraged to quarantine and test; if they end up testing positive within 14 days of the original case, we can link the two positive individuals, and consider this link a unique disease surveillance artifact of contact tracing. Multiple contacts can be linked to one case, and multiple cases can be linked to one contact.

Second, we observe the artifacts of *mandated reporting* and associated outbreak investigation as exposure events categorized by location type and link all pairs within an event that are also within 14 days of each other, regardless of whether the outbreak investigation team ultimately determined that they met the definition of a confirmed outbreak. We have enough events from *schools*, *workplaces*, *jails*, and *LTCFs* to evaluate them as distinct location types of reporting, while events from other settings like shelters are insufficiently numerous to analyze on their own. We consider these disease surveillance strategies to be active given their reliance on trained personnel to consider cases one by one.

Disease surveillance can also include passive retrieval of links from other information gathered, such as a shared *home address* (collected upstream via testing) or shared *employer*, *school*, or nonresidential *location history* (collected via survey or interview). These are passive in the sense that they are derived not from direct linkage of individuals via active staff effort, but rather from post hoc matching of patient-provided information, and are for the most part not currently used to identify potential outbreaks (though they may fill gaps in ongoing investigations).

Patient address is present in data from the lab that performs the COVID-19 test, but it must be geocoded to produce a location usable for spatiotemporal scans and to prevent matching failure due to different ways of writing the same address. In order to protect patient privacy and prevent leakage of information via a third-party API, we perform geocoding offline using fuzzy record linkage to a comprehensive table of addresses in SCC, provided by private data vendor Melissa, which includes latitude and longitude. The query address is parsed to extract house number, street name, unit, and postal code. We first search for exact matches, and then for addresses that do not have an exact match in Melissa, we use the *fuzzyjoin* R package with a custom matching function. Our matching algorithm finds an exact, unit-level match for 82.1% of cases in the study period and finds at least a match with the same house/building number for 85.0%. The resulting matched address is used to identify cases at the same address, and the latitude and longitude are used as input to SaTScan’s spatiotemporal scan algorithm.

Same school links require an exact match on three fields filled in as part of the informational interview or survey: city location of the school, type of school (elementary, middle, or high school), and the school name. Fields are lightly cleaned to remove punctuation and remote locations. Same supervisor links require at least one match out of three fields: supervisor name, supervisor phone number, or supervisor e-mail address. Location histories are unique object types in CalCONNECT which specify the start and end date range for a patient visiting a specific location, uniquely identified by location name and address. We impose an additional time-based constraint that two cases must have start and end date ranges within seven days of overlapping one another (i.e., the start of the second case’s location history must be within seven days of the end of the first case’s location history), in addition to the 14-day window for episode date. Location histories were used both passively by contact tracers during interviews and actively by investigators to record cases who were present at an exposure event but believed to have been exposed to SARS-CoV-2 off-site; in other words, investigated cases linked through location histories were expected to be less-plausible transmission links. Available location history information was ultimately sparse because of a variety of factors, but has the potential to be more substantively collected with future technology and protocol improvements.

Altogether, these data allow us to identify proposed links between cases for an array of different strategies. We use these proposed links to measure the effectiveness of each disease surveillance strategy at identifying plausible SARS-CoV-2 transmission links. While we assess the informational value of each strategy independently, we also assess set differences of two strategies, such as removing same address links from the set of contact tracing links to assess inter-household spread.

### Quantification and statistical analysis

#### Spatiotemporal clustering

We make use of SaTScan, a software package which implements spatial, temporal, and space-time scan statistics for disease surveillance and analysis.^37^ We use the retrospective space-time scan statistic, developed by Kulldorff, which tests whether observations of COVID-19 are uniformly distributed among units over space and time, adjusting for underlying spatial and temporal inhomogeneity.^40^ The scan uses a “moving cylinder”, with the base and height corresponding to the spatial and temporal dimensions respectively. As the cylinder “slides” across space and time, SaTScan uses statistical tests to measure whether the case prevalence in the area of interest is significantly higher than the baseline process would suggest. SaTScan identifies spatiotemporal “hotspots”, so mere proximity (e.g., three cases at the same address) is not sufficient to be flagged; cases must exhibit statistically significant concentrations. SaTScan offers several baseline models, and the choice of model largely corresponds to the type of data available: Bernoulli for 0–1 data corresponding to infected and uninfected people, Poisson for case data combined with knowledge of the underlying population at risk, and a space-time permutation model for case data only. Since CalCONNECT only gives us information about COVID-19 infections, we use the space-time permutation model.^41^

SaTScan is highly configurable. Some parameters, such as the maximum radius of the scan cylinder in space and its maximum duration in time, do not have obvious defaults, and their values may substantially affect the sort of clusters that SaTScan produces. We want to test SaTScan with a “good” set of parameters, but also avoid overfitting to the study data and biasing the result. In order to pick reasonable parameters, we conduct a randomized grid search over possible values of the radius (between 0.05 and 5 km) and the time-duration (7, 14, or 21 days). Each of the n = 250 trials uses a random combination of radius and time-duration, and a randomly chosen contiguous two-month subset of the case data. We measure the number of false positive links (found by SaTScan but ruled out by genomic data) against true positive links (found by SaTScan and not ruled out by genomic data). We evaluate only links that are not within a household, since links within a household can be discovered without SaTScan. These results are shown in Figure S5 in the SI, grouped by radius quintile and time-duration, with each point corresponding to a trial. The steeper the slope of that line, the higher the “cost” in false positives as more true plausible transmission links are discovered. The results suggest that a smaller radius is likely to perform best, and that increasing the radius would identify more plausible transmission links only at a substantial loss of precision. As a result, we select 0.1 km for the radius parameter. Given this choice of radius, the time-duration appears to be inconsequential, and so we select 14 days, which is most in line with estimates of the typical COVID-19 incubation period (5 days) plus typical duration of contagiousness (10 days).^42^^,^^43^ Figure S6 in the SI compares the informational value of the SaTScan configuration chosen for the main analysis (14 days, 0.1 km) with an alternative configuration that performed worse on precision and recall (7 days, 2 km). As expected, the looser configuration identifies more overall links but at the cost of lower informational value on average (between the average informational value of long-term care facility and jail reporting).

#### Genomic data processing

To implement our method for providing biological ground-truth to COVID-19 surveillance strategies, we gather a large sample of whole-genome sequences from the County’s genomic data platform, Terra, as well as from GISAID. Because of the limited search functionality of GISAID, the initial set of genomes downloaded is by necessity overly broad: 152,337 sequences available on GISAID up to February 24, 2022, accessible via the key resources table. We then match these genomes to metadata from the County linking them to cases in CalCONNECT. We discard any sequences not linked to a known COVID-19 case in SCC. Sequences only became available at scale starting in the middle of 2021, and sequencing was limited by laboratory capacity, which meant that, especially during surge periods, there were often backlogs, and not all cases could be sequenced.

Of the 67,374 sequenced SCC cases available from May 1 to December 31, 2021, not all are suitable for analysis, since a sequence with high missingness appears trivially similar to many other sequences. Sequences are aligned using the open-source Nextclade command-line interface, using the standard 2019 Wuhan strain as a reference sequence.^44^ Nextclade provides several metrics for aligned sequences, including an overall quality category (“good”, “mediocre”, “bad”) and a count of missing base pairs. We discard all “bad” sequences except those that are only categorized as “bad” due to private mutations, since newer variants tended to be overwhelmingly categorized as “bad” for this reason. We also, following SCC laboratory protocol, discard all sequences missing more than 850 out of the 29,903 base pairs in the SARS-CoV-2 genome, so that all included samples have >97% breadth of coverage relative to Wuhan-1. We match the remaining high-quality sequences to the CalCONNECT records in our study period, leaving us with a sample of 10,131 high-quality sequences (3,067 from Terra and 7,064 from GISAID) linked to case records at a 15% match rate. We show case counts from CalCONNECT over time in Figure S7 in the SI, along with the number and fraction (rate) of cases matched to high quality sequences.

#### Sequencing balance

Genomic sequencing is not randomly assigned to individuals who test positive with COVID-19. Whether a case is sequenced, and whether we have access to that sequence, depends on the lab performing the testing and the priorities of the County in selecting which of an abundant pool of genomes to sequence given its limited resources. Though our method of measuring informational value is as a proportion of sequenced edges, it is still conceivable that non-random sequencing could bias the results. The availability of sequences for a given disease surveillance strategy also affects our confidence in the estimate of that strategy’s informational value: fewer sequences results in a wider confidence interval.

Table S1 in the SI shows the sequencing rate by a variety of demographic factors of interest, with 95% confidence intervals modeled as a binomial proportion. There is no statistically significant difference in sequencing by gender, as self-reported. Cases with unknown factors (unknown gender, race or ethnicity) appear less likely to be sequenced. The difference between non-Hispanic/Latino and Hispanic/Latino sequencing rates is also substantial; in our view, this may be a result of the County’s targeting of outreach to the Spanish-speaking community. We suspect that Hispanic/Latino patients were more likely to get a COVID-19 test at a free County-run test site given their geographic placement, and as a result, their test samples were more likely to be sequenced by the County and find their way into our dataset.

Table S2 in the SI shows the sequencing rate by disease surveillance strategy, also with a 95% binomial proportion confidence interval. A case “belongs” to a given disease surveillance strategy if it appears in at least one link identified by that strategy. There are substantial differences here, for which we can only offer conjectures. Some differences may reflect the County’s priorities when it comes to sequencing (e.g., choosing to sequence cases from jails more often). They may also reflect the same pattern as in Table S1: since the County testing sites that were more likely to be sequenced were geographically concentrated in Hispanic neighborhoods, then certain disease surveillance strategies, like contact tracing, that were also geographically targeted would appear to have positively correlated sequencing rates. COVID-19 hotspots in Hispanic neighborhoods would also explain why SaTScan cases are sequenced at a higher rate, since such spatiotemporal methods, by design, identify locations with unusually high case rates.

#### Informational value

We define the informational value of each disease surveillance strategy as the fraction of sequenced links surfaced by the strategy that are biologically plausible according to genomic sequence data. Because not all pairs of cases are sequenced, we can only estimate informational value based on the sample of sequenced cases. We restrict the analysis to proposed pairs of cases whose episode dates are within 14 days of one another.

To determine whether the genomic data support a proposed transmission link, we measure the genomic distance between sequenced pairs of cases. A straightforward way to measure the similarity of two sequences is the number (or proportion) of observed substitutions (i.e., the Hamming distance). However, this does not account for the uncertainty introduced by missing or ambiguous sites. Bootstrapping is a statistical method used to estimate the sampling distribution of a random variable, based only on the observed data, by resampling from the observed data with replacement.^45^ Felsenstein (1985) applies this idea to phylogenetic trees, using it to estimate branch support values (i.e., confidence values on clades) by resampling genome sites with replacement. These bootstrap samples are used to construct a new genome, and then a phylogenetic tree is constructed from the bootstrapped genome.^39^ By doing this many times, and observing how often a clade appears, one can calculate a support value for the clade. In our setting, we are interested in evolutionary distances rather than clade support values, but these distances can also change across bootstrap iterations, as missing and mismatched sites can be resampled or omitted. Therefore, we apply phylogenetic bootstrapping, in combination with ordinary bootstrap resampling over cases, to calculate non-parametric confidence intervals on our measure of informational value.

For each bootstrap iteration, we first perform a resampling (with replacement) from the 29,903 sites of the SARS-CoV-2 genome, which may repeat or omit sites from the original sequence, and construct a phylogenetic tree with evolutionary distances based on maximum likelihood. We consider two sequences to constitute a plausible transmission link if their distance (i.e., expected number of substitutions) is fewer than two base pairs. The expected number of substitutions is the estimated substitution rate (observed mismatches divided by the number of non-missing sites) multiplied by the total number of sites. This quantity differs across bootstrap iterations because: (a) *mismatched* sites may be repeated or omitted, which changes the numerator of the substitution rate; and (b) *missing* sites may be repeated or omitted, which changes the denominator of the substitution rate. We conduct our analysis in Terra, which uses IQ-TREE to build phylogenetic trees.^38^ By supplying a command-line argument, we can specify that the tree be constructed using the phylogenetic bootstrap, in which case all *n* bootstrapped trees are outputted along with the consensus tree. Each bootstrapped tree results in slightly different evolutionary distances, which in turn affects which pairs are considered plausible transmission links, which incorporates uncertainty about similarity between strains into our downstream estimates of informational value.

Only a minority of cases in CalCONNECT are sequenced, and of those that are sequenced, not all are of high quality. Informational value is thus estimated from the sample of the population of all COVID-19 cases that have high quality sequences. To quantify the sampling uncertainty of this method, on top of the uncertainty from missing sites in genomes, we also apply bootstrapping over cases. To do this, for each bootstrap iteration, we resample all 67,374 CalCONNECT cases with replacement. When a case is selected multiple times, its corresponding proposed links count multiple times. For example, if case A is sampled three times, and case B is sampled one time, then link A-B is counted three times. If case B was instead sampled twice, then link A-B is counted six times. When a case is not sampled, then its edges do not count at all. Resampling cases rather than links is faithful to the data generating process, in which cases are the unit selected to be sequenced in a laboratory or not, and links can exist between any pair of cases that was chosen to be sequenced.

Finally, for each iteration, we group all sequenced pairs of cases that are identified by a disease surveillance strategy and calculate that strategy’s informational value, equal to the fraction of proposed sequenced case pairs, counted using the weights from case resampling, that are also plausible transmission links, according to the bootstrapped phylogenetic tree. We repeat this process for 100 trials using parallelization, given one bootstrap alone requires nearly 24 h of computation. From the bootstrap trials, we obtain a distribution of estimates for the informational value of each disease surveillance strategy, allowing us to calculate empirical confidence bounds and conduct statistical tests to determine whether the yield of a given strategy is significantly higher than the yield of another. We report p values when comparing two disease surveillance strategies, defined as the proportion of the 100 trials in which their ranking reverses, compared to their ranking based on average informational value.

#### Robustness to different thresholds for plausible transmission

In the main analysis, we consider two sequences to constitute a plausible transmission link if their distance (i.e., expected number of substitutions) is fewer than two base pairs. We selected this threshold in close consultation with SCC epidemiological staff. Given that SARS-CoV-2 is a relatively small genome with a relatively low mutation rate, and that the higher our threshold, the more pairs of sequences would be considered to be “transmission links,” we concluded that our choice of possible thresholds was practically limited to small integer numbers. Empirically, we note that over half of all pairs of sequences, across 100 phylogenetic bootstrap iterations, differ by fewer than 20 base pairs, while more than a quarter of all pairs differ by fewer than three base pairs.

Nonetheless, given the possibility that our results are sensitive to our selected threshold, we also recompute our main results from Figure 1 using different base pair thresholds, from one to five. The robustness check can be seen in Figure S8 in the SI. We see that, as expected, higher base pair thresholds are associated with higher informational values. We also see that, in general, the rank order of disease surveillance strategies does not significantly change, which suggests that our approach is relatively robust to the exact method of defining transmission and related uncertainty, and is surfacing robust insights about the relative differences in yield between distinct disease surveillance strategies. As notable exceptions, Jail Reporting (and, as a result, All Mandated Reporting) and Same Occupation Location are the disease surveillance strategies most sensitive to different threshold definitions, which suggests that our main results may underestimate their informational value, but not in ways that fundamentally change our key takeaways.

#### Robustness to alternative uncertainty measures

The main results reported in Figure 1 use a methodology that accounts for both uncertainty from missing sites (via phylogenetic bootstrapping), and uncertainty from sampling (via case bootstrapping). We compare this “double-bootstrap” approach to some simpler approaches, as illustrated in Figure S9 in the SI, to understand the relative contributions of two bootstrapping steps to the overall uncertainty and to see if the added complexity is worthwhile.

##### Phylogenetic bootstrapping only

In this approach, we ignore sampling uncertainty, and only consider uncertainty from missing sites in sequence data. This is not a recommended approach since sampling uncertainty should be accounted for, but it helps to understand the relative contribution of phylogenetic uncertainty. These intervals are often (but not always) the narrowest intervals, implying that sampling uncertainty contributes more heavily.

##### Pairwise Distances, binomial confidence interval

Pairwise distances are calculated directly by simply counting the number of mismatched sites (also called single-nucleotide polymorphisms, or SNPs) between pairs of strains. This ignores any uncertainty from missing sites. In this approach, we also forgo bootstrapping cases, and instead treat the fraction of proposed edges that are plausible transmission links as a *binomial proportion*. We assume each link proposed by a disease surveillance strategy is plausible with probability *p*, estimate *p* as equal to the number of plausible links divided by the number of proposed edges, and estimate the 95% confidence interval with the Clopper-Pearson exact method. This is a simpler way to consider sampling uncertainty but a less accurate representation of the data-generating process. Given that cases are the actual unit sampled, links are not independent of one another: knowing that link A-B and link B-C are sequenced implies that link A-C is also sequenced. Thus, we think it makes more sense to treat cases as the unit of consideration, as we do in the next two approaches.

##### Pairwise distances, case-bootstrap confidence interval

As in the previous approach, we ignore sequence missingness, and only consider sampling uncertainty. Instead of treating links as randomly sampled for sequencing from the population, we treat cases as the unit of interest, and quantify uncertainty by resampling cases from the population. Then, the sampled cases are used downstream to reweight the links. When a case is absent because it isn’t sampled, its links are not counted. When a case is sampled multiple times, all its links are reweighted so that they count proportionally to how many times the case was sampled.

##### Phylogenetic bootstrapping, case-bootstrap confidence interval

This is the methodology used for the main analysis. While all four uncertainty methodologies yield a similar rank ordering of informational value of disease surveillance strategies, we ultimately select this methodology to incorporate the most conservative confidence intervals into our comparisons across disease surveillance strategies.
