# Supplementary material for: Leveraging genomic sequencing data to evaluate disease surveillance strategies
Source: iScience. 2023 Nov 19;26(12):108488. doi: 10.1016/j.isci.2023.108488 (PMC10711492; doi:10.1016/j.isci.2023.108488)
Supplement: Document S1. Figures S1–S9 and Tables S1 and S2 [file mmc1.pdf]

iScience, Volume *26*

Supplemental information

Leveraging genomic sequencing data

to evaluate disease surveillance strategies

Benjamin Anderson, Derek Ouyang, Alexis D'Agostino, Brandon Bonin, Emily Smith, Vit Kraushaar, Sarah L. Rudman, and Daniel E. Ho

Figure S1. Pairwise overlap of disease surveillance strategies, related to Figure 1

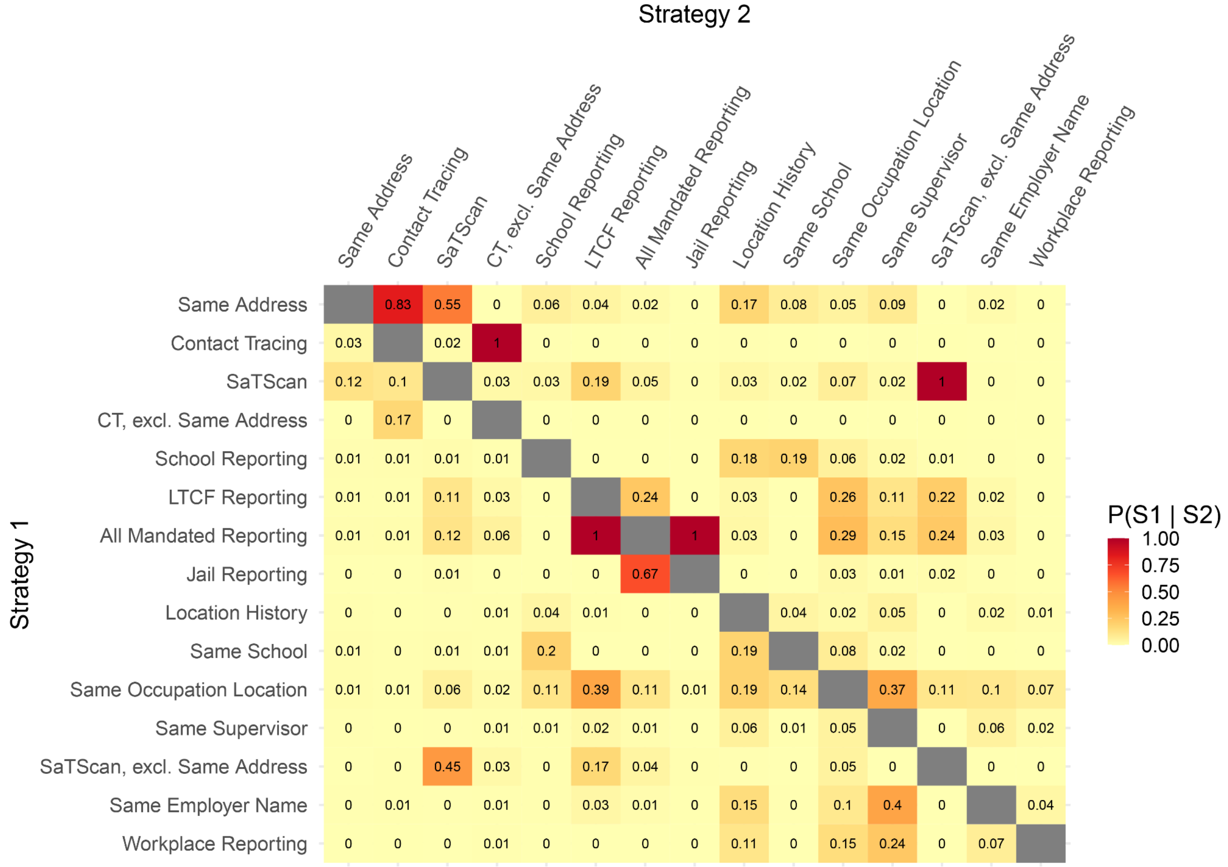

Figure S1. Author's analysis of Santa Clara County CalCONNECT and genomic sequencing data from May 1 to December 31, 2021. Each cell's value and color represents the probability that a link is found by Strategy 1, conditional on being found by Strategy 2. *Contact Tracing*: identified as close contacts by contact tracing. *Same Address*: shared home address. *SaTScan*: assigned to the same cluster by spatiotemporal analysis. *All Mandated Reporting*: linked through mandated reporting in a high-risk setting; *School*, *Long-Term Care Facility*, *Jail*, and *Workplace Reporting* are sufficiently numerous and sequenced to also be presented on their own. The following five strategies are based on matching results from the case interview or survey. *Location History*: matching location visited within the same time period. *School*: same school name, school type, and school location. *Occupation Location*: same workplace location, usually an address. *Supervisor*: same supervisor name, phone number, or email. *Employer Name*: same employer name. CT = Contact Tracing; LTCF = Long-Term Care Facility.

Figure S2. Pairwise performance comparisons of disease surveillance strategies, related to Figure 1

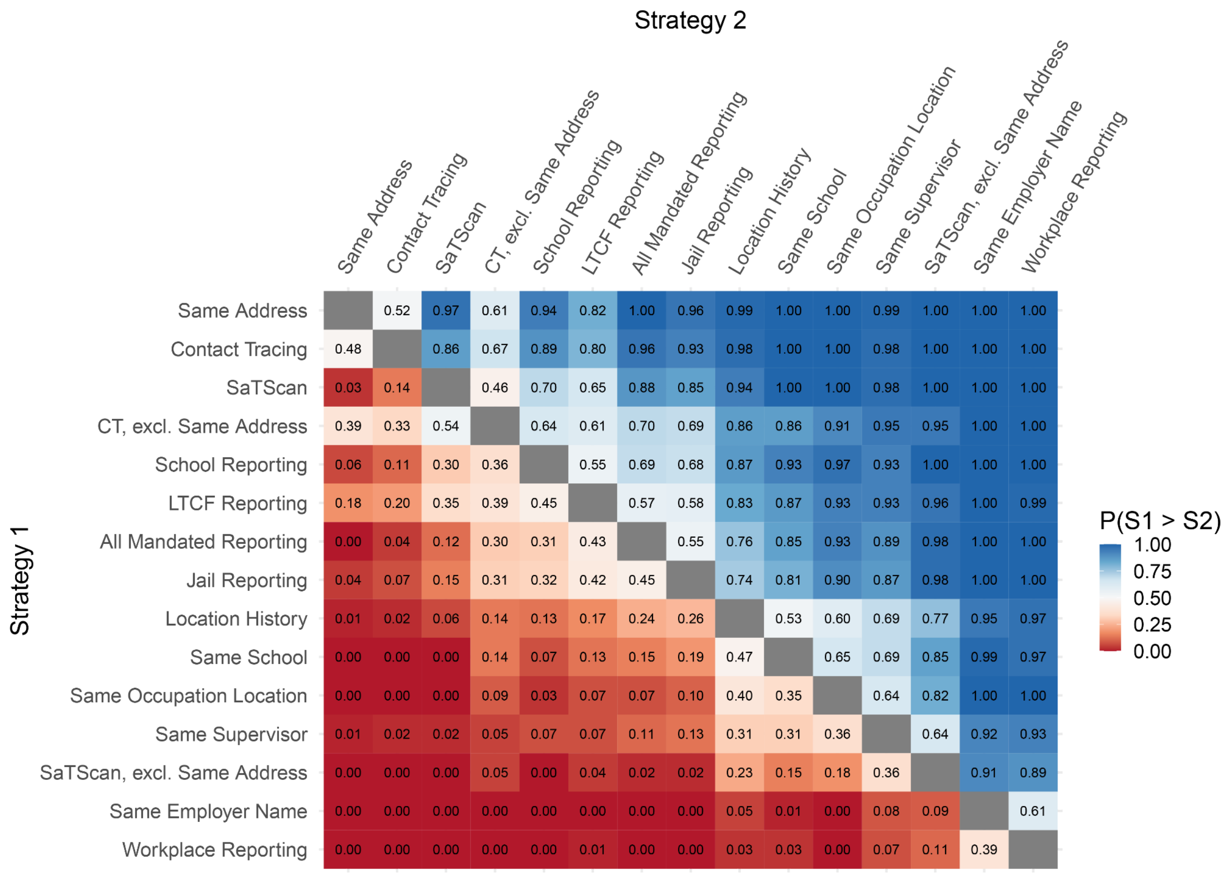

Figure S2. Author's analysis of Santa Clara County CalCONNECT and genomic sequencing data from May 1 to December 31, 2021. Each cell's value and color represents the percentage of 100 bootstrap trials in which Strategy 1's informational value is greater than Strategy 2's informational value. *Contact Tracing*: identified as close contacts by contact tracing. *Same Address*: shared home address. *SaTScan*: assigned to the same cluster by spatiotemporal analysis. *All Mandated Reporting*: linked through mandated reporting in a high-risk setting; *School*, *Long-Term Care Facility*, *Jail*, and *Workplace Reporting* are sufficiently numerous and sequenced to also be presented on their own. The following five strategies are based on matching results from the case interview or survey. *Location History*: matching location visited within the same time period. *School*: same school name, school type, and school location. *Occupation Location*: same workplace location, usually an address. *Supervisor*: same supervisor name, phone number, or email. *Employer Name*: same employer name. CT = Contact Tracing; LTCF = Long-Term Care Facility.

Figure S3. Informational value of disease surveillance strategies by genome source, related to Figure 1

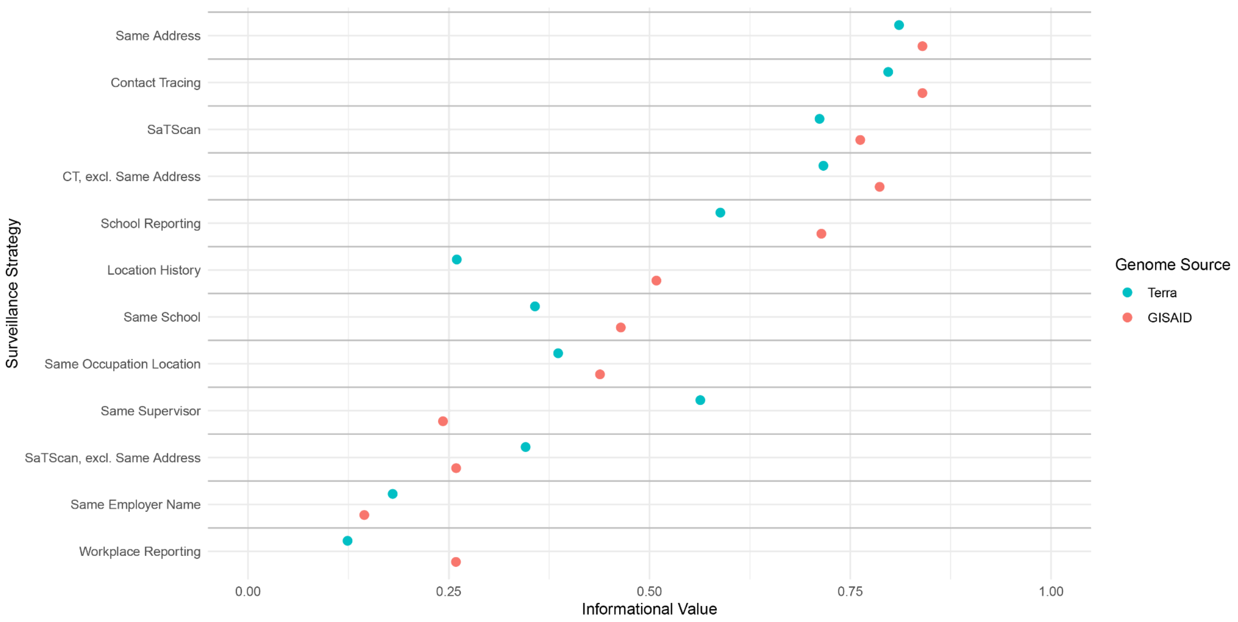

Figure S3. Author's analysis of Santa Clara County CalCONNECT and genomic sequencing data from May 1 to December 31, 2021. Each point represents the average informational value out of 100 bootstrap trials for a given surveillance strategy. Each color distinguishes a subset of cases from a different genome source: Terra or GISAID. *Contact Tracing*: identified as close contacts by contact tracing. *Same Address*: shared home address. *SaTScan*: assigned to the same cluster by spatiotemporal analysis. *All Mandated Reporting*: linked through mandated reporting in a high-risk setting; *School*, *Long-Term Care Facility*, *Jail*, and *Workplace Reporting* are sufficiently numerous and sequenced to also be presented on their own. The following five strategies are based on matching results from the case interview or survey. *Location History*: matching location visited within the same time period. *School*: same school name, school type, and school location. *Occupation Location*: same workplace location, usually an address. *Supervisor*: same supervisor name, phone number, or email. *Employer Name*: same employer name. CT = Contact Tracing; LTCF = Long-Term Care Facility.

Figure S4. Informational value of disease surveillance strategies by genome source (scatter plot), related to Figure 1

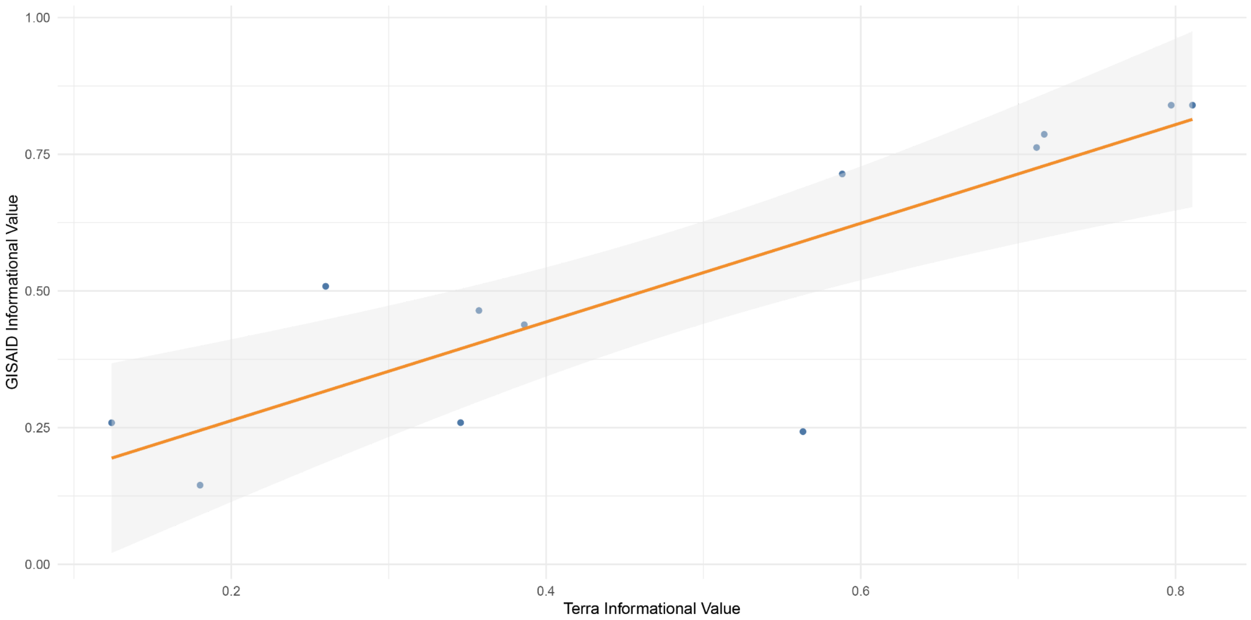

Figure S4. Author's analysis of Santa Clara County CalCONNECT and genomic sequencing data from May 1 to December 31, 2021. Each point represents a surveillance strategy, where the x-axis represents the average informational value out of 100 bootstrap trials for case sequences Terra or GISAID. The linear regression line is plotted with a 95% confidence interval. *Contact Tracing*: identified as close contacts by contact tracing. *Same Address*: shared home address. *SaTScan*: assigned to the same cluster by spatiotemporal analysis. *All Mandated Reporting*: linked through mandated reporting in a high-risk setting; *School*, *Long-Term Care Facility*, *Jail*, and *Workplace Reporting* are sufficiently numerous and sequenced to also be presented on their own. The following five strategies are based on matching results from the case interview or survey. *Location History*: matching location visited within the same time period. *School*: same school name, school type, and school location. *Occupation Location*: same workplace location, usually an address. *Supervisor*: same supervisor name, phone number, or email. *Employer Name*: same employer name. CT = Contact Tracing; LTCF = Long-Term Care Facility.

Figure S5. SaTScan hyperparameter tuning results, related to STAR Methods

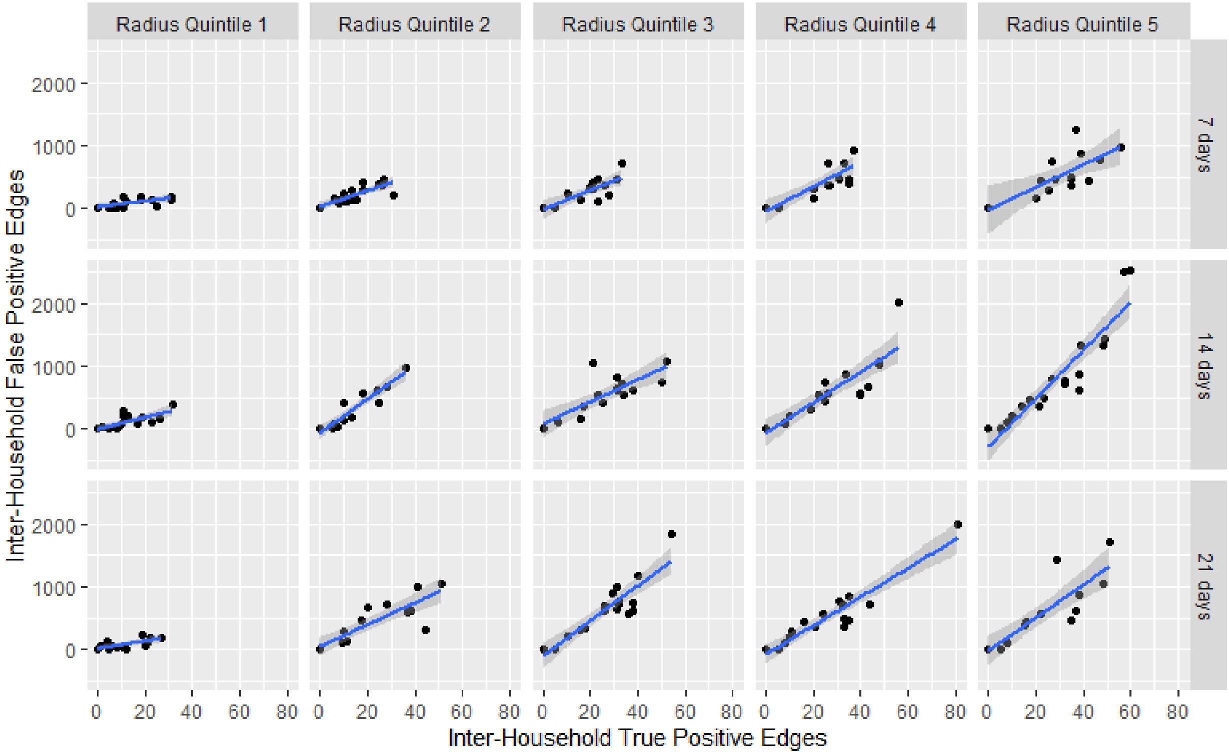

Figure S5. Author's analysis of Santa Clara County CalCONNECT and genomic sequencing data from May 1 to December 31, 2021. Each point corresponds to one of 250 SaTScan trials on random two-month subsets of case data and random possible values of the radius (between 0.05 and 5km) and the time-duration (7, 14, or 21 days), grouped by radius quintile and time-duration. The y-axis is the number of false positive inter-household edges in the trial (found by SaTScan but ruled out by genomic data), and the x-axis is the number of true positive inter-household edges (found by SaTScan and not ruled out by genomic data). Linear regression lines are plotted with 95% confidence intervals.

Figure S6. Information value of SaTScan configurations, related to STAR Methods

Disease Surveillance Strategy	Informational Value	95% CI	# Seq. Links
Contact Tracing	0.83	(0.67, 0.94)	161
SaTScan (0.1km, 14d)	0.75	(0.66, 0.82)	729
SaTScan (2km, 7d)	0.63	(0.54, 0.73)	1181

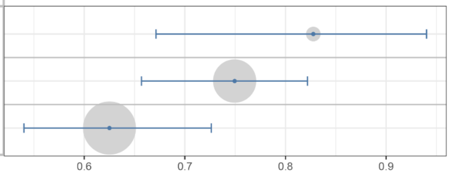

Figure S6. Author’s analysis of Santa Clara County CalCONNECT and genomic sequencing data from May 1 to December 31, 2021. For each strategy, we show: the informational value (proportion of sequenced case pairs which are plausible transmission links according to whole-genome sequence data); the 95% confidence interval on informational value; and the number of proposed links which are sequenced. The first two rows reflect results from Table 1, where SaTScan is configured with a radius of 0.1km and a time-duration of 14 days. The third row presents an alternative configuration of SaTScan with a radius of 2km and a time-duration of 7 days.

Figure S7. Cases, sequences, and sequencing rate by week, related to STAR Methods

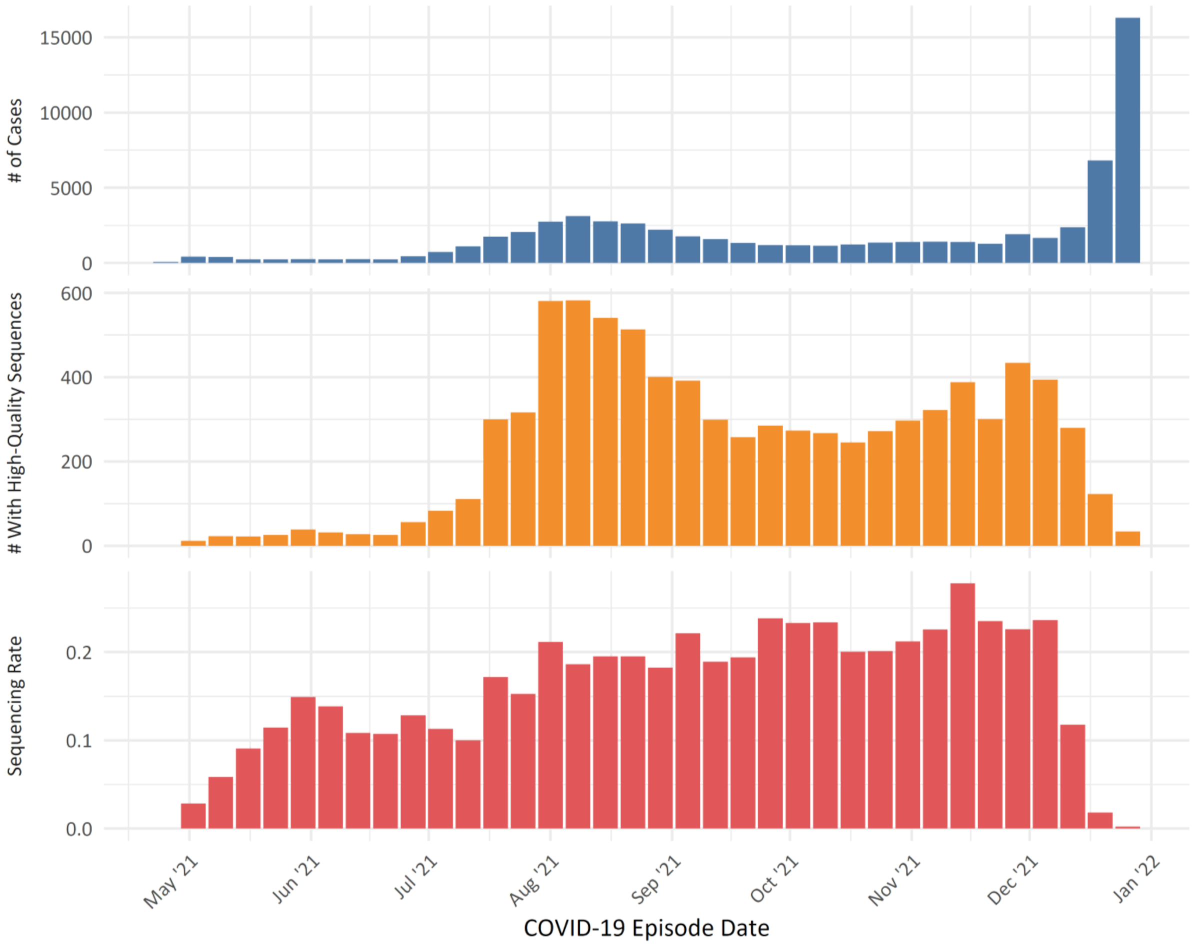

Figure S7. Author's analysis of Santa Clara County CalCONNECT and genomic sequencing data from May 1 to December 31, 2021.

Table S1. Sequencing rate by demographic factors, related to STAR Methods

Demographic Group	# Cases	# Sequences	Sequencing Rate	95% CI
All	67,374	10,131	0.150	(0.148, 0.153)
Male	33,025	5,082	0.154	(0.150, 0.158)
Female	32,840	4,885	0.149	(0.145, 0.153)
Other/Unknown Gender	1,509	164	0.109	(0.093, 0.125)
White	19,271	2,737	0.142	(0.137, 0.147)
Asian	12,362	2,283	0.185	(0.178, 0.192)
Black	1,930	297	0.154	(0.138, 0.171)
Hawaiian / Pacific Islander	766	136	0.178	(0.151, 0.206)
American Indian / Alaska Native	626	148	0.236	(0.204, 0.272)
Other / Two or More Races	16,654	2,488	0.149	(0.144, 0.155)
Unknown Race	15,765	2,042	0.130	(0.124, 0.135)
Hispanic / Latino	21,260	4,423	0.208	(0.203, 0.214)
Not Hispanic / Latino	32,339	4,793	0.148	(0.144, 0.152)
Other/Unknown Ethnicity	13,775	915	0.066	(0.062, 0.071)

Table S1. Author's analysis of Santa Clara County CalCONNECT and genomic sequencing data from May 1 to December 31, 2021. Gender, race, and ethnicity information is available via CalCONNECT for each case. 95% confidence intervals are modeled as a binomial proportion.

Table S2. Sequencing rate by disease surveillance strategy, related to STAR Methods

Disease Surveillance Strategy	# Cases	Sequencing Rate	95% CI	% Hispanic / Latino
All	67,374	0.150	(0.148, 0.153)	31.6%
Contact Tracing	1,877	0.291	(0.271, 0.313)	55.6%
Same Address	27,409	0.190	(0.185, 0.195)	32.7%
SaTScan	1,934	0.263	(0.243, 0.283)	53.4%
Contact Tracing, excl. Same Address	339	0.242	(0.197, 0.291)	58.4%
School Reporting	1,172	0.130	(0.111, 0.150)	31.3%
Long-Term Care Facility Reporting	705	0.104	(0.082, 0.128)	28.3%
Jail Reporting	424	0.238	(0.198, 0.282)	46.2%
All Mandated Reporting	1,389	0.150	(0.132, 0.171)	33.8%
Location History	782	0.202	(0.174, 0.232)	45.5%
Same School	2,205	0.155	(0.140, 0.171)	40.7%
Same Occupation Location	2,641	0.122	(0.109, 0.135)	30.6%
Same Supervisor	567	0.164	(0.134, 0.197)	41.7%
SaTScan, excl. Same Address	906	0.264	(0.235, 0.294)	55.4%
Same Employer Name	2,388	0.166	(0.151, 0.181)	37.7%
Workplace Reporting	1,197	0.099	(0.082, 0.117)	29.4%

Table S2. Author's analysis of Santa Clara County CalCONNECT and genomic sequencing data from May 1 to December 31, 2021. A case "belongs" to a given disease surveillance strategy if it appears in at least one link identified by that strategy. 95% confidence intervals are modeled as a binomial proportion. Ethnicity information is available via CalCONNECT for each case. *Contact Tracing*: identified as close contacts by contact tracing. *Same Address*: shared home address. *SaTScan*: assigned to the same cluster by spatiotemporal analysis. *All Mandated Reporting*: linked through mandated reporting in a high-risk setting; *School*, *Long-Term Care Facility*, *Jail*, and *Workplace Reporting* are sufficiently numerous and sequenced to also be presented on their own. The following five strategies are based on matching results from the case interview or survey. *Location History*: matching location visited within the same time period. *School*: same school name, school type, and school location. *Occupation Location*: same workplace location, usually an address. *Supervisor*: same supervisor name, phone number, or email. *Employer Name*: same employer name.

Figure S8. Informational value of disease surveillance strategies by base pair threshold, related to STAR Methods

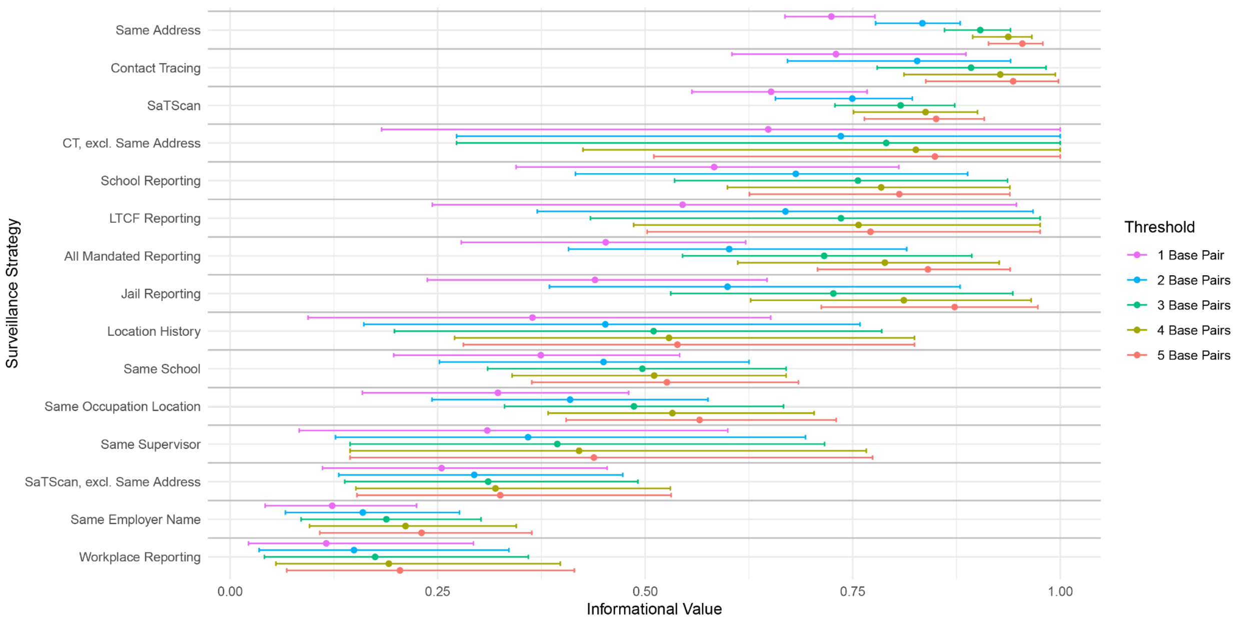

Figure S8. Author’s analysis of Santa Clara County CalCONNECT and genomic sequencing data from May 1 to December 31, 2021. The points represent informational value (proportion of sequenced case pairs which are plausible transmission links according to whole-genome sequence data); the bars represent the 95% confidence interval on informational value. The five colors shown represent different definitions of plausible transmission, from fewer than one base pair distance to fewer than five base pairs. Fewer than two base pairs, in blue, represents our main results. *Contact Tracing*: identified as close contacts by contact tracing. *Same Address*: shared home address. *SaTScan*: assigned to the same cluster by spatiotemporal analysis. *All Mandated Reporting*: linked through mandated reporting in a high-risk setting; *School*, *Long-Term Care Facility*, *Jail*, and *Workplace Reporting* are sufficiently numerous and sequenced to also be presented on their own. The following five strategies are based on matching results from the case interview or survey. *Location History*: matching location visited within the same time period. *School*: same school name, school type, and school location. *Occupation Location*: same workplace location, usually an address. *Supervisor*: same supervisor name, phone number, or email. *Employer Name*: same employer name. CT = Contact Tracing; LTCF = Long-Term Care Facility.

Figure S9. Informational value of disease surveillance strategies by uncertainty methodology, related to STAR Methods

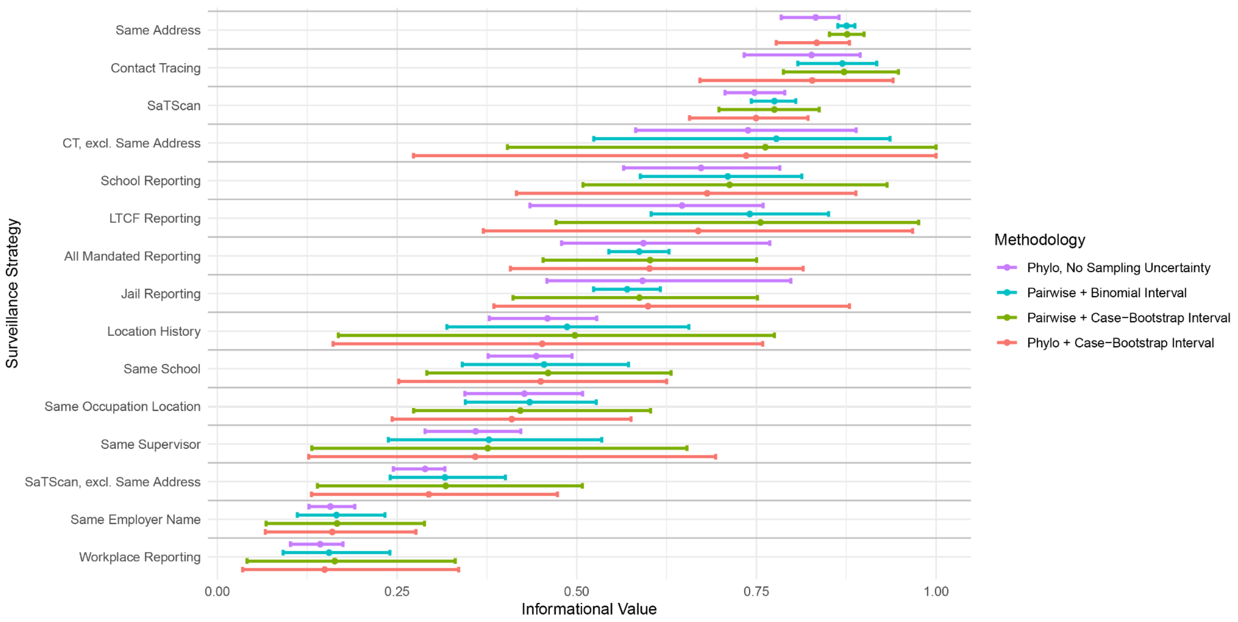

Figure S9. Author's analysis of Santa Clara County CalCONNECT and genomic sequencing data from May 1 to December 31, 2021. The points represent informational value (proportion of sequenced case pairs which are plausible transmission links according to whole-genome sequence data); the bars represent the 95% confidence interval on informational value. Four uncertainty methodologies are shown in different colors: (1) phylogenetic bootstrapping with no case sampling uncertainty; (2) pairwise distances with binomial case confidence intervals; (3) pairwise distances with bootstrap case confidence intervals; and (4) phylogenetic bootstrapping with bootstrap case confidence intervals, as used in the main analysis. *Contact Tracing*: identified as close contacts by contact tracing. *Same Address*: shared home address. *SaTScan*: assigned to the same cluster by spatiotemporal analysis. *All Mandated Reporting*: linked through mandated reporting in a high-risk setting; *School*, *Long-Term Care Facility*, *Jail*, and *Workplace Reporting* are sufficiently numerous and sequenced to also be presented on their own. The following five strategies are based on matching results from the case interview or survey. *Location History*: matching location visited within the same time period. *School*: same school name, school type, and school location. *Occupation Location*: same workplace location, usually an address. *Supervisor*: same supervisor name, phone number, or email. *Employer Name*: same employer name. CT = Contact Tracing; LTCF = Long-Term Care Facility.
